# Active learning for accuracy enhancement of semantic segmentation with CNN-corrected label curations: Evaluation on kidney segmentation in abdominal CT

**DOI:** 10.1038/s41598-019-57242-9

**Published:** 2020-01-15

**Authors:** Taehun Kim, Kyung Hwa Lee, Sungwon Ham, Beomhee Park, Sangwook Lee, Dayeong Hong, Guk Bae Kim, Yoon Soo Kyung, Choung-Soo Kim, Namkug Kim

**Affiliations:** 1 Asan Medical Institute of Convergence Science and Technology, University of Ulsan College of Medicine, Asan Medical Center, Seoul, 05505 Republic of Korea; 2 Department of Convergence Medicine, University of Ulsan College of Medicine, Asan Medical Center, 88 Olympic-ro 43 Gil, Songpa-gu Seoul, 05505 South Korea; 3 Department of Radiology, University of Ulsan College of Medicine, Asan Medical Center, 88 Olympic-ro 43 Gil, Songpa-gu Seoul, 05505 South Korea; 4 ANYMEDI Inc., 388-1 Pungnap2-dong, Songpa-gu Seoul, South Korea; 5 Department of Health Screening and Promotion Center, Asan Medical Center, 88 Olympic-ro 43 Gil, Seoul, 05505 South Korea; 6 Department of Urology, University of Ulsan College of Medicine, Asan Medical Center, 88 Olympic-ro 43 Gil, Seoul, 05505 South Korea

**Keywords:** Kidney, Computer science

## Abstract

Segmentation is fundamental to medical image analysis. Recent advances in fully convolutional networks has enabled automatic segmentation; however, high labeling efforts and difficulty in acquiring sufficient and high-quality training data is still a challenge. In this study, a cascaded 3D U-Net with active learning to increase training efficiency with exceedingly limited data and reduce labeling efforts is proposed. Abdominal computed tomography images of 50 kidneys were used for training. In stage I, 20 kidneys with renal cell carcinoma and four substructures were used for training by manually labelling ground truths. In stage II, 20 kidneys from the previous stage and 20 newly added kidneys were used with convolutional neural net (CNN)-corrected labelling for the newly added data. Similarly, in stage III, 50 kidneys were used. The Dice similarity coefficient was increased with the completion of each stage, and shows superior performance when compared with a recent segmentation network based on 3D U-Net. The labeling time for CNN-corrected segmentation was reduced by more than half compared to that in manual segmentation. Active learning was therefore concluded to be capable of reducing labeling efforts through CNN-corrected segmentation and increase training efficiency by iterative learning with limited data.

## Introduction

Image segmentation is a fundamental component of medical image analysis^1–3^. It is a prerequisite for computer aided detection and provides quantitative information for treatment, surgical planning, and 3D printing in medicine, etc^4,5^. Recent advances in deep learning, such as the emergence of fully convolutional networks (FCN) have enabled the training of models for semantic segmentation tasks^6^. Especially, 3D U-Net, which has a contracting path and a symmetric expanding path, has been proven to be effective for 3D medical image segmentation^7^. Some authors have proposed novel cascaded architectures such as segmentation-by-detection networks and cascaded 3D FCN to improve segmentation performance using region proposal network prior to segmentation^8–12^.

However, the aforementioned methods currently face serious obstacles, such as the difficulty in acquiring sufficient and high-quality training data owing to the scarcity of medical image datasets, variation of human labels, and high labeling efforts and costs. Deep learning architectures require large amounts of input data to train the network and avoid overfitting. However, real-world medical images are usually limited, and only trained medical experts can annotate data in most cases. In particular, image segmentation in the cases of rare diseases and complex abdominal structures, such as renal cell carcinoma (RCC) and ureters of the kidney, labeling is relatively more difficult owing to high anatomical variability. To alleviate the burden of manual annotation, active learning frameworks were introduced in several studies^13–20^. Most authors proposed active learning to build generalizable models with the smallest number of additional annotations^13–19^. Generally, active learning aims to select the most informative queries or areas to be labeled among a pool of unlabeled or uncertain samples. Some authors applied interactive learning framework incorporating CNNs into a bounding box and scribble-based segmentation for generalizability to previously unseen object classes^20^.

In this study, we propose another active learning framework to reduce labeling efforts as well as increase efficiency with limited training data of medical images. The purpose of this study is to verify if segmentation accuracy and annotation efficiency can be improved through the use of active learning.

## Results

### Segmentation results

Table 1 presents the DSC of five subclasses in each stage and the difference of DSC between stages. The average values of DSC for the five subclasses were increased with the completion of each stage. Among the aforementioned subclasses, parenchyma segmentation has the highest DSC and the lowest standard deviation (SD) values, while RCC demonstrated values of the lowest DSC and the highest SD. In addition, the final segmentation results in the last stage was superior when compared with the nnU-Net using our dataset as described in Table 2.

**Table 1:** The Dice similarity coefficient (DSC) evaluation.

Class	DSC (%)			P-value	
	Stage 1	Stage 2	Stage 3	Stage 1 and 3	Stage 2 and 3
Artery	44.30 ± 10.12	66.03 ± 8.65	63.56 ± 12.86	0.372	0.704
Vein	72.60 ± 10.39	77.94 ± 8.42	75.00 ± 13.40	0.837	0.873
Ureter	48.04 ± 12.02	60.43 ± 7.66	60.56 ± 8.45	0.088	0.655
Parenchyma	95.83 ± 0.56	96.12 ± 0.72	96.27 ± 0.70	0.697	0.772
Renal Cell Carcinoma	11.47 ± 14.63	46.76 ± 30.42	52.55 ± 34.57	0.239	0.131
Total	54.45 ± 30.34	70.65 ± 21.30	71.07 ± 21.65	0.252	0.330

**Table 2:** Comparison of segmentation time between manual and CNN-corrected segmentation.

Time	Manual segmentation	CNN-corrected segmentation
Artery	41 m 8 s	22 m
Vein	35 m 1 s	23 m
Ureter	24 m 23 s	5 m
parenchyma	26 m 26 s	18 m 6 s
RCC	22 m 8 s	5 m
Total	149 m 6 s	73 m 6 s

### Comparison of time and root-mean-square between manual and CNN-corrected segmentation

**Figure 1:**
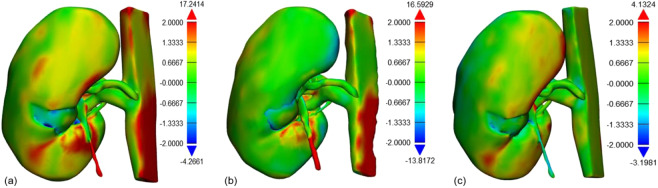
Results of part comparison analysis in 3D models between (**a**) manual and CNN segmentation, (**b**) CNN and CNN-corrected segmentation, and (**c**) manual and CNN-corrected segmentation.

The results of the comparison of segmentation time for the five substructures between manual and CNN-corrected segmentation are listed in Table 3. CNN-corrected segmentation decreased the time for artery segmentation by 19 min 8 s, and that of the vein, ureter, parenchyma, and RCC by 12 m 1 s, 19 m 23 s, 8 m 20 s, and 17 m 8 s, respectively. According to the results, the overall segmentation time was reduced by 76 min, which is more than half of the time required in manual segmentation. Except for the initial loading of the package, the CNN segmentation took less than 1 s per case. The differences between manual, CNN, and CNN-corrected segmentation by quantitative evaluation in 3D models are presented in Table 3 and Fig. 1. The results of CNN-corrected segmentation are observed to highly correspond with those of manual segmentation, while those of CNN segmentation do not.

**Table 3:** Root-mean-square (RMS) evaluation from 3D modeling.

Comparison	RMS (mm)
Manual and CNN segmentation	2.22 ± 2.06
CNN and CNN-corrected segmentation	2.77 ± 2.77
Manual and CNN-corrected segmentation	0.86 ± 0.80

## Discussion

Medical image segmentation is a tedious and labor-intensive task. Although the recent developments in CNNs has enabled easy and fast segmentation, segmentation of complex abdominal organs with insufficient medical images is still a challenge. In this study, we used a cascaded 3D U-Net with an active learning framework for semantic segmentation of RCC and fine substructures of the kidney. Consequently, the network demonstrated an improved performance in several respects. First, active learning was found to improve the network by iterative learning with limited data for training. The segmentation accuracy increased over the stages, and overall performance was reasonable compared with other state-of-the-art segmentation network. Furthermore, it was able to reduce the effort required for creating new ground truths from scratch. Just modification from the CNN segmentation was more efficient and timesaving as well as less variable compared to manual annotation.

In this study, the authors used cascaded 3D U-Net architecture for coarse region detection followed by fine region segmentation and trained this architecture with active learning. Recent 3D U-Nets have achieved impressive results in medical image segmentation, thereby becoming the most popular networks for semantic segmentation^7^. However, some authors developed this network by combining detection modules in a cascading manner^8–12^. Tang *et al*. proposed a cascade framework comprising a detection module using VGG-16 model followed by a segmentation module^9^. Roth *et al*. also demonstrated a second-stage FCN in a cascading manner that focused more on the target boundary regions^10^. In their studies, the cascaded network showed a superior performance compared to a single 3D U-Net. To validate the performance of proposed method, we compared it with the recent competitive network, nnU-Net^21^. This network had achieved excellent performance in KiTS19 challenge with the ability to dynamically adapt to the details of the datasets. However, the result of nnU-Net applied to our dataset was inferior than ours. Some of the reasons might be explained by insufficient pre-processing for a dataset and that the tuning process for training data had been far different in our dataset.

Active learning framework has been introduced in several studies for segmentation on histology data to reduce annotation effort by making judicious suggestions on the most effective annotation areas^13–20^. Yang *et al*. presented a deep active learning framework by combining FCNs and active learning. In this framework, an annotation suggestion approach directed manual annotation efforts to the most effective annotation areas^14^. Lubrano di Scandalea *et al*. also proposed a similar framework for the segmentation of myelin sheath from histology data, wherein they employed Monte-Carlo Dropout to assess model uncertainty and select samples to be annotated for the next iteration^17^. In this study, active learning was used for the new ground truths to be segmented preliminary to manual correction and the network to be iteratively trained with limited data, instead of suggesting the most effective annotation area. Our model demonstrated superior performance during the later stages. The DSC value in each stage was increased with the completion of each stage, which means that the network improved by iterative learning and the use of additional labels.

CNN-corrected segmentation was found to be more effective when compared to manual segmentation. The former can be conducted in a much easier and faster way than manual segmentation. Considering that labeling is fundamental but extremely labor-intensive, which makes it difficult to initiate deep learning, this model can be considered to be a useful alternative in this regard. In addition, human labels are not always constant in the segmentation process due to intra-human and inter-human variabilities. Active learning frameworks may reduce this uncertainty by increasing collaboration with the deep learning algorithm, leading to enhanced accuracy.

However, there are several limitations to this study. As the error must be less than 2 mm when the results of segmentation are applied to other medical technologies such as 3D printing, virtual reality and augmented reality, the proposed framework is not suitable for direct application without manual correction. Further efforts to increase accuracy by increasing the amount of training data and utilizing superior networks to resolve ambiguities^22,23^, may be required. In addition, further validation using more data and comparison with other segmentation networks should be performed to verify its stability and efficiency.

## Conclusion

Active learning in semantic segmentation was proven to cause reduction of labeling effort and time using CNN-corrected segmentation and also increase training efficiency through iterative learning with very limited training data.

## Methods

**Figure 2:**
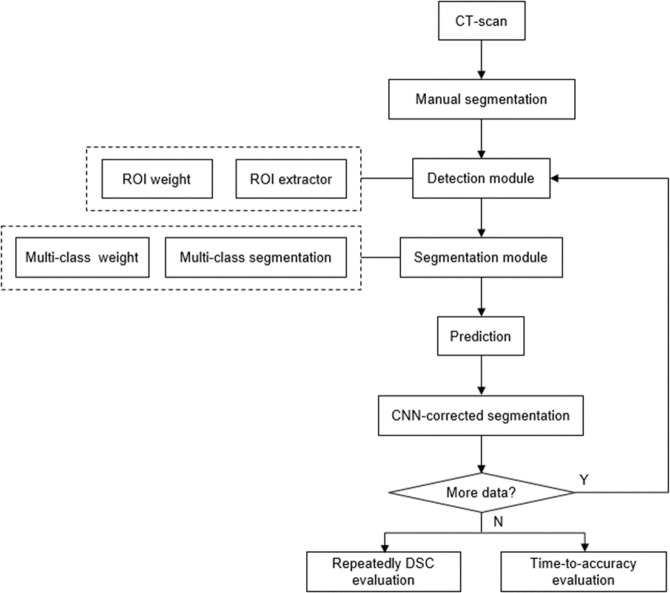
Workflow of active learning framework.

First, we trained the model using a cascaded 3D U-Net with exceedingly small amount of training data and the corresponding ground truths were generated by manual labeling at the initial stage. The cascaded architecture was designed to improve segmentation performance using region proposal network (RPN) prior to segmentation within the available memory of the graphics processing unit (GPU). Second, the results of the additional data through the trained network were manually corrected instead of creating new ground truths from scratch. This step is called convolutional neural network (CNN)-corrected segmentation, as depicted in Fig. 2. Third, all the data initially used and newly added were used again for subsequent training. Figure 2 illustrates the overall process of the active learning framework for segmentation.

### Dataset acquisition

A total of 50 kidneys, of which 30 had RCCs and 20 were normal kidneys, from 36 patients in abdominal computer tomography (CT)-scans (Sensation 16, Siemens Healthcare) with slice thickness of 1–1.25 mm each were utilized. There were four types of phases in the CT scans; the non- contrast, renal cortical, renal parenchymal and renal excretory phases. We used the renal cortical phase, which enhanced the arteries, and classified kidneys into five subclasses such as artery, vein, ureter, parenchyma with medulla, and RCC in the case of kidneys with RCC. We excluded kidneys that included any cysts or stones. The Institutional review board for human investigations at Asan Medical Center (AMC) approved the retrospective study with a waiver of informed consent. The imaging data were de-identified in accordance with the Health Insurance Portability and Accountability Act privacy rule.

### Cascaded 3D U-Net

**Figure 3:**
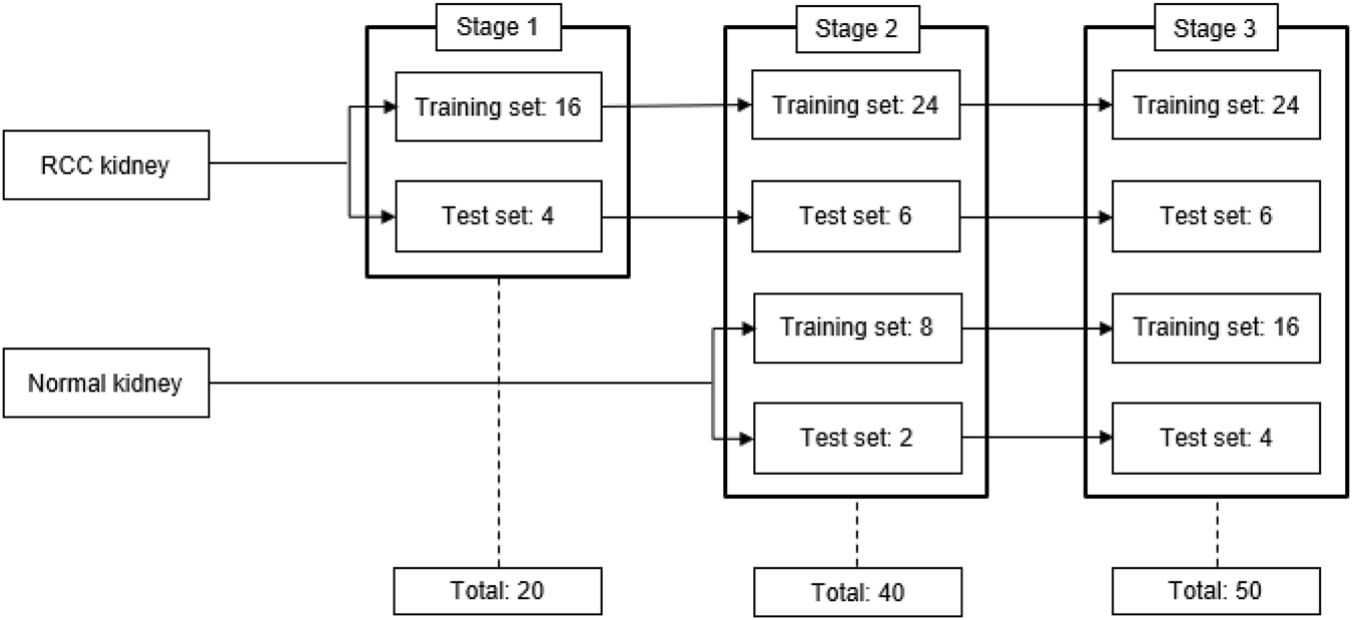
Data numbers in each stage of active learning.

We used a cascaded 3D U-Net architecture, which replaced the first RPN in the study of Tang *et al*.^9^ into 3D U-Net^7^, which shows superior accuracy in the detection of the region of interest (ROI) of the kidney in the abdominal CT. The 3D U-Net (Fig. 3) can be divided into two main sections. The left side reduces the number of dimensions and the right side extends to the original number of dimensions. The two sides consist of convolution and up or down sampling layers. Down-sampling was used by max pooling (3 × 3 × 3). The prominent feature of 3D U-Net is that it has a concatenation function to the left and to the right. The concatenation results lead to an improved segmentation by preventing the loss of information.

Cascaded 3D U-Net was separately trained in an end-to-end manner. The ROI was determined as cuboidal bounding box around the kidney after first U-Net module. Subsequently, second U-Net module for final segmentation was trained to make the mask for 5 subclasses of the kidney. Each input image was added with a Gaussian noise. The errors were calculated using the dice similarity coefficient (DSC), same as that in Eq. (1). The loss function, denoted by dice loss (DL), was defined as Eq. (2) in each 3D U-Net. V_GT_ and V_CNN_ were defined as the volume of ground truth and CNN segmentation, respectively.

1 $${\rm{DSC}}\,({{\rm{V}}}_{{\rm{GT}}},{{\rm{V}}}_{{\rm{CNN}}})=\frac{2|{{\rm{V}}}_{GT}{\cap }^{}{{\rm{V}}}_{CNN}|}{|{{\rm{V}}}_{GT}|+|{{\rm{V}}}_{CNN}|}$$

2 $${\rm{DL}}=1-\,\frac{2|{{\rm{V}}}_{GT}{\cap }^{}{{\rm{V}}}_{CNN}|}{|{{\rm{V}}}_{GT}|+|{{\rm{V}}}_{CNN}|}$$

### Active learning

**Figure 4:**
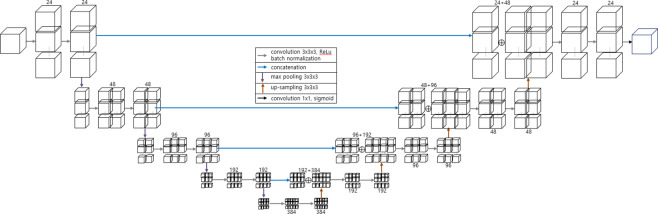
3D U-Net architecture.

In stage I, 5 subclasses of the 20 kidneys including artery, vein, ureter, parenchyma, and RCC were manually delineated as ground truths for initial training. After stage I, the ground truths of new data for next stage were prepared by manual correction on the results from CNN segmentation, which was referred to as CNN-corrected segmentation. In stage II, 16 kidneys in the previous stage were reused for training, with new data including 8 kidneys with RCCs and 8 normal kidneys shown as Fig. 4. After stage II, the results of CNN segmentation for the new data were manually amended for the next stage, as in stage I. Finally, in stage III, 40 kidneys were used for training, and 10 kidneys were used for testing. The results of all the aforementioned stages were used for accuracy evaluation. The manual and CNN-corrected segmentations were conducted using Mimics software (Mimics; Materialise, Leuven, Belgium).

### Experimental settings

The model was executed in Keras 2.2.4 with Tensorflow 1.14.0 backend and trained with a GPU of NVIDIA GTX 1080 Ti. Our cascade method generally requires a large number of epochs in both steps. In the first stage, the training was saturated at about 150 epochs, due to the small number (N = 20) of datasets. The second and third stages required 300 epochs due to increased numbers (N = 40, and 50) of datasets. In addition, Adam optimizer with learning rate of 10−5, weight decay of 0.0005, a momentum of 0.9, the training loss as average dice coefficient loss, batch size of 1 was used. For testing the overfitting of the model, the difference of overall DSC accuracies between validation and test datasets of the final model were 6.17 which demonstrated that this model is not overfit.

### Evaluation and statistical analysis

To observe whether the performance of the network improves or not through active learning, we investigated the DSC in each stage and compared them using the paired t-test between stages 1 and 3, and stages 2 and 3, using SPSS software (version 25.00; IBM). In addition, to evaluate the effect of the proposed method, we compared it with more recent network, no-new-U-Net (nnU-Net) introduced by Isensee *et al*.^21^. This network won the first place in Kidney Tumor Segmentation Challenge (KiTS19) on Medical Image Computing and Computer Assisted Intervention Society (MICCAI) 2019. We also validated the CNN-corrected segmentation based on accuracy and consumption time for evaluating the labeling efficiency. We converted the results of manual, CNN, and CNN-corrected segmentation to 3D models to compare the accuracy. The comparison was performed based on points in the surface, using quantitative root-mean-square (RMS) values in the 3-matic software (3-matic; Materialise, Leuven, Belgium). 17,650 points were used for comparing 3D models among manual and CNN segmentations, and manual and CNN-corrected segmentations. For comparison of CNN segmentation with CNN-corrected segmentation, 26,471 points were calculated. The calculation for RMS is the same as that of Eq. (3), where *x* is a difference between corresponding points in the two models, and *n* is the total number of points.

3 $${\rm{RMS}}=\sqrt{\frac{1}{n}({x}_{1}^{2}+{x}_{2}^{2}+{x}_{3}^{2}+\ldots +{x}_{n}^{2})}=\sqrt{\frac{1}{n}\mathop{\sum }\limits_{k=1}^{n}{x}_{k}^{2}}$$

## Data Availability

The datasets generated during and/or analyzed during the current study are available from the corresponding author on reasonable request.
