# Author Correction: Active learning for accuracy enhancement of semantic segmentation with CNN-corrected label curations: Evaluation on kidney segmentation in abdominal CT

**DOI:** 10.1038/s41598-022-08496-3

**Published:** 2022-03-16

**Authors:** Taehun Kim, Kyung Hwa Lee, Sungwon Ham, Beomhee Park, Sangwook Lee, Dayeong Hong, Guk Bae Kim, Yoon Soo Kyung, Choung-Soo Kim, Namkug Kim

**Affiliations:** 1 Asan Medical Institute of Convergence Science and Technology, University of Ulsan College of Medicine, Asan Medical Center, Seoul, 05505 Republic of Korea; 2 Department of Convergence Medicine, Asan Medical Center, University of Ulsan College of Medicine, 88 Olympic-ro 43 Gil, Songpa-gu, Seoul, 05505 South Korea; 3 Department of Radiology, University of Ulsan College of Medicine, Asan Medical Center, 88 Olympic-ro 43 Gil, Songpa-gu, Seoul, 05505 South Korea; 4 ANYMEDI Inc., 388-1 Pungnap2-dong, Songpa-gu, Seoul, South Korea; 5 Department of Health Screening and Promotion Center, Asan Medical Center, 88 Olympic-ro 43 Gil, Seoul, 05505 South Korea; 6 Department of Urology, University of Ulsan College of Medicine, Asan Medical Center, 88 Olympic-ro 43 Gil, Seoul, 05505 South Korea

Correction to: *Scientific Reports* 10.1038/s41598-019-57242-9, published online 15 January 2020

The original version of this Article contained an error in the spelling of the author Kyung Hwa Lee which was incorrectly given as Kyunghwa Lee.

The original Article has been corrected.

